# Revision Lumbar Spine Surgeries: An Early Career Neurosurgery Experience

**DOI:** 10.7759/cureus.57371

**Published:** 2024-04-01

**Authors:** Bilal Khan, Syed Mansoor Shah, AbdUllah Khan, Hubab Ali, Atta Ullah, Ihsan Ullah, Usman Haqqani, Riaz Uliqbal

**Affiliations:** 1 Neurosurgery, Medical Teaching Institution-Lady Reading Hospital (MTI-LRH), Peshawar, PAK; 2 Neurosurgery, Queen Elizabeth Hospital, UHB Trust, Birmingham, GBR; 3 Clinical Neurosciences, Royal Infirmary of Edinburgh, Edinburgh, GBR; 4 Neurological Surgery, Medical Teaching Institution-Lady Reading Hospital (MTI-LRH), Peshawar, PAK; 5 Neurosurgery, Qazi Hussain Ahmed Medical Complex, Nowshera, PAK

**Keywords:** post-operative adhesions, spinal-fusion, listhesis, recurrent lumbar disk herniation, neurosurgery, early career experience, revision lumbar spine surgery

## Abstract

Background: The aims and ambitions of a surgeon in the early years of his professional career are to make a good reputation by providing excellent patient outcomes and avoiding complex and difficult surgeries. Revision lumbar spine surgeries (RLSSs) pose a significant challenge in terms of surgical management, as the moribund anatomy increases the risk of complications, adding to an unlikely outcome.

Objective: We conducted this study to determine the clinical indications and outcomes of RLSSs performed by an early career neurosurgeon.

Materials and methods: This cross-sectional study was conducted after approval from the hospital's ethical committee, and data was collected in late December of 2022 and early January 2023, from retrospective records for a single early career neurosurgeon. A form was filled with each patient’s data, such as age, gender, time since surgery, indication for surgery, operative findings, types of surgery performed, etc. All variables were noted for the patient and were further categorized, based on the clinical records, into many sub-categories.

Results: Almost 400 lumbar spine surgeries were performed by the surgeon, and about 45 (11.25%) were revision surgeries, and the full record was available for 42 surgeries. These patients' ages ranged from 22 to 70 years, and the mean age was about 46.74*±*13.29 SD. The common symptoms leading to revision surgeries were numbness and pain in 17 (40.5%) patients each; common per-operative findings were recurrent disc in eight patients (19%), infection in nine patients (21.4%), and fibrosis/adhesions in 16 (38.1%); most common surgeries performed were diskectomy in 11 (26.2%) and diskectomy plus release of adhesions in 12 (28.6%); complications occurred in 14 (33%), and good to excellent outcomes was recorded in 29 (69%) cases.

Conclusion: RLSSs are difficult compared to first-time lumbar spine surgeries, and the moribund anatomy predisposes to complications, and better shall be dealt with great care and, at the minimum, shall be embarked upon as a team.

## Introduction

Lumbar spine surgery constitutes a major bulk of neurosurgical and orthopedics spine surgery’s operative burden, and about one million back-related surgeries are performed annually in the United States, constituting about 6.52% of all operating room procedures [1]. Yet the rate of revision spine surgeries among these cases hovers around 10.2-23%, making it a significant figure to consider [2,3]. Revision lumbar spine surgeries (RLSSs) could be for various reasons like recurrent disk, surgical site infection in the form of diskitis or superficial wound infection, adjacent segment disease, instability, and low back pain; these surgeries are technically challenging and have inconstant results compared to a naïve surgery [4,5]. The time between the initial surgery and the recurrent surgery could be as short as a few days to as long as more than a decade [3]. Many risk factors have been identified for recurrent lumber spine surgery, like age, gender, traumatic event, smoking status, active lifestyle, and higher body mass index [4,6]. The most widely studied cause for RLSSs is recurrent lumbar disk herniation (rLDH), and the other factors and indications like stenosis and instability are least studied [4-6]. RLSSs pose many challenges for the operative surgeon because of the moribund anatomy and scar tissues, making dissection and identification of neural structures very difficult, hence increasing the risk of complications like dural tears and nerve injuries [3-5]. Also, the outcome and type of surgical interventions are changed, as there may be a need for an additional procedure like fusion if instability has ensued pre- or per-operatively [4,7].

Because of these reasons, RLSSs pose a formidable challenge not only for a recently qualified consultant but also for an experienced neurosurgeon. In the early years of consultancy, many surgeons are wary of performing any procedures independently and about 64% of the surgical residency graduates opt for sub-specialty training, according to a study [8], in these contexts embarking on a revision surgery of the lumbar spine is very challenging job for an early career neurosurgeons. This study aimed to determine the primary indications in these surgeries, the type of surgeries performed, the complications encountered, and the relief of patient's symptoms in the immediate postoperative period within a cohort of patients re-operated by an early career neurosurgeon.

## Materials and methods

This cross-sectional study was conducted after approval from the Ethical Committee of Lady Reading Hospital, Peshawar (vide letter no. 179/LRH/MTI), and data was collected in late December 2022 and early January 2023. Retrospectively, records were checked for all patients admitted and operated on during the last five years by a single neurosurgeon in his early years of consultancy. Only patients with RLSSs were included, and those who underwent first-time surgery were excluded from the study. A form was made and filled with each patient's records like age, gender, time since surgery, indication for surgery, operative findings, types of surgery performed, any complications encountered, and early outcome at the first follow-up visit, as per the clinical records of the patient (Table 1). All variables were noted for the patient and were further categorized into many sub-categories based on the clinical records. Results for relief of symptoms were construed from the frequency of clinical visits and patient complaints. Interrelationships between various variables like type of surgery, indications for surgery, gender, and the early outcome were determined, and any complications encountered, like a dural tear or nerve injury resulting in post-operative neural deficit, were also noted. Patients with incomplete records were also excluded from the study. The group contained subcategories of a patient initially operated on by the same surgeon and those who were not.

**Table 1:** Proforma questionnaire for the study. CES: Cauda equina syndrome

Gender	Male/Female
Age in years	Number in digits
Symptoms	Present(y)/Absent(n)
Weakness	y/n
CES	y/n
Numbness	y/n
Pain	y/n
Per-operative findings	Present (y)/Absent (n)
Infection	y/n
Instability	y/n
Listhesis	y/n
Fibrosis	y/n
Recurrent disk	y/n
Type of surgery performed	Yes (y)/No (n)
Diskectomy	y/n
Diskectomy with release of adhesions	y/n
Decompression/Laminectomy	y/n
Debridement	y/n
Fusion	y/n
Adjacent segment decompression	y/n
Previous surgery by the same surgeon (yes/no)	Yes/No
Complications (n)	Present (y)/Absent (n)
Dural rent	y/n
Nerve injuries	y/n
Cauda Equina syndrome	y/n
Infection	y/n
Years (level) of experience in surgeries performed	Yes (y)/No (n)
` Year 1	y/n
Year 2	y/n
Year 3	y/n
Year 4	y/n
Year 5	y/n
Early outcome	Present (y)/Absent (n)
Excellent	y/n
Good	y/n
Satisfactory	y/n
No improvement	y/n
Deteriorated	y/n

Patients were examined in the outpatient department or at the private practice clinic in the hospital premises. They were admitted after having necessary investigations like magnetic resonance imaging (MRI) of the lumbar spine and other needful investigations like flexion-extension X-rays of the lumbar spine. Routine and special investigations for co-morbidities were also done, and a detailed history and examination were taken after admission. Operative procedures were explained to the patients, and informed consent was obtained from all the patients. All the patients were operated on in a single center setting (Lady Reading Hospital, Peshawar) through midline incision using the same operative protocols. Monopolar cautery was used for the dissection of the spinal muscles, and bone was taken with the help of Kerrison rongeur. Spinal procedures were done according to every patient's requirements, and the wound was closed in layers using absorbable sutures for the muscles, fascia, and subcutaneous tissues. The skin was completed using non-absorbable sutures like Prolene® 2/0. Patients were put on antibiotics for five days and were assessed fortnightly in the outpatient clinic.

Data was entered and analyzed using IBM SPSS Statistics for Windows, Version 23 (Released 2015; IBM Corp., Armonk, New York, United States) and was expressed into tables and charts. Frequency and percentages were used for qualitative variables like gender and types of surgery, while mean was used for quantitative variables like age and time since surgery. Tests for correlation between various variables were performed to determine their significance, especially between the years of experience and the complications, previous surgery by the same surgeon, and the early outcome.

## Results

Data was retrieved from the charts and files of the patients and the hospital software and was reviewed for all cases of spine surgery operated during the desired period. The surgeon performed 900 procedures, including nearly 400 lumbar spine surgeries for various etiologies like trauma, disk problems, stenosis, spine instability, etc. About 45 (11.25%) were revision surgeries, and the entire record was available for 42 surgeries. These patients' ages ranged from 22 to 70 years, and the mean age was about 46.74±13.29 SD. There were 25 males and 17 females, with a male-to-female ratio approaching 1.5:1. The minimum duration since the first surgery was one month, the maximum was 120 months, and the mean duration was about 43.45 months. The most common symptoms leading to a repeat surgery were pain and numbness/claudication; other symptoms were weakness and Cauda Equina Syndrome. Per-operative findings were also recorded; the main signs were pus, fibrosis, instability, and frank listhesis. The type of surgeries performed, whether previous surgery was done by the same surgeon, the list of complications and their relative frequencies, and early outcomes' results are all tabulated in Table 2.

**Table 2:** Results and relative frequencies of various variables.

Variable	Frequency/percentage
Gender (m/f)	25/17
Age in years (median±SD)	46.74±13.29 STD
Symptoms	n(%)
Weakness	6(14.3)
CES	1(2.4)
Numbness	17(40.5)
Pain	17(40.5)
Per-operative findings	n(%)
Infection	9(21.4)
Instability	7(16.7)
Listhesis	2(4.8)
Fibrosis	16(38.1)
Recurrent disk	8(19)
Type of surgery performed	n(%)
Diskectomy	11 (26.2)
Diskectomy with release of adhesions	12 (28.6)
Decompression/Laminectomy	5 (11.9)
Debridement	7 (16.7)
Fusion	6 (14.28)
Adjacent segment decompression	1 (2.4)
Previous surgery by the same surgeon (yes/no)	7/35 (16.7%/83.3%)
Complications (n)	14 (33.4%)
Dural rent	7 (16.7)
Nerve injuries	2 (4.8)
Cauda Equina syndrome	1 (2.4)
Infection	4(9.6)
Years of experience-wise surgeries performed	n(%)
` Year 1	12 (28.6)
Year 2	12 (28.6)
Year 3	6 (14.3)
Year 4	3 (7.1)
Year 5	9 (21.4)
Early outcome	n (%)
Excellent	3 (7.1)
Good	26 (61.90)
Satisfactory	10 (23.8)
No improvement	2 (4.76)
Deteriorated	1(2.38)

Revision surgeries were performed mainly in the first two years and the last year of early consultancy. The surgical load was reduced in the years 3 and 4 due to the COVID-19 pandemic. Revision surgeries for operations performed by the same surgeon previously were highest in the first year (6/7) and were lowest in the fifth year (1/7). The mean duration for surgeries performed initially by the same surgeon was 3.28±1.38SD months; for surgeries not performed by the same surgeon, it was 3.74±0.61SD months. There was no significant difference between the means after conducting an independent sample t-test (n=42, p= .41); equal variances were not assumed.

**Figure 1:**
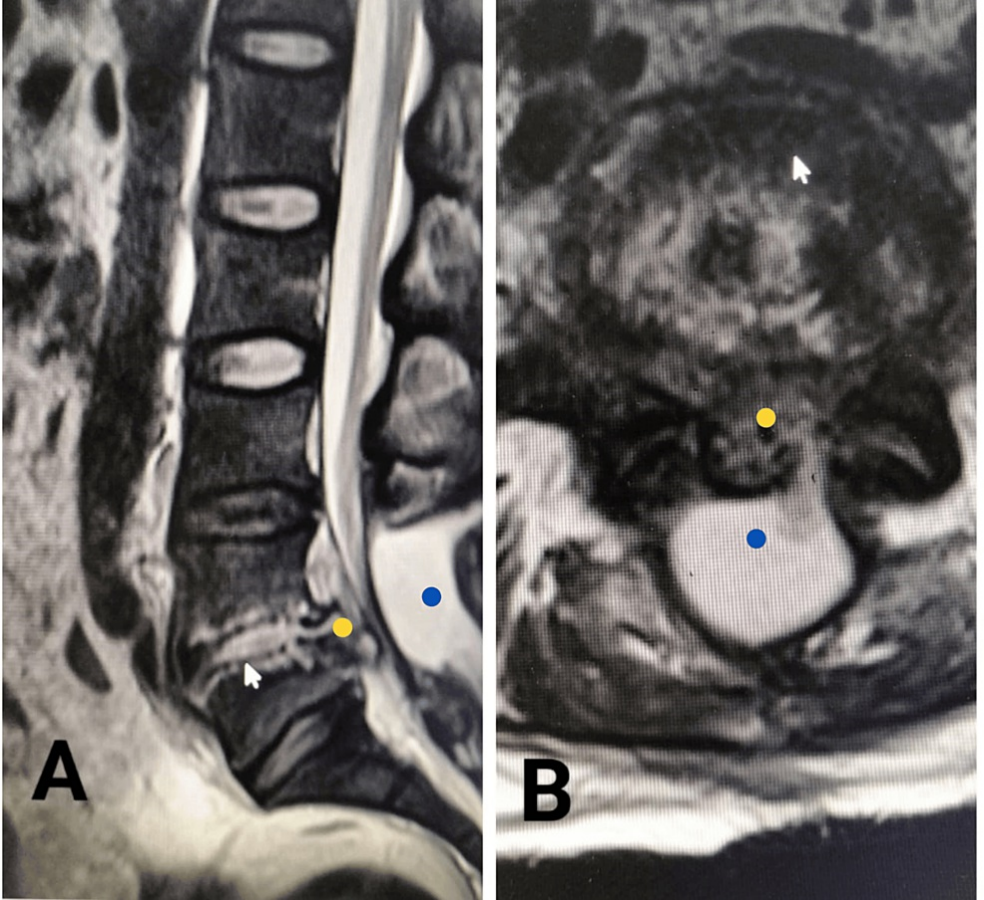
MR-T2WI of the lumbar spine of a patient who was operated on four months back for spine surgery and has been bed-ridden since that. The patient could not move and walk due to pain. The sagittal (A) and axial (B) images show marked changes in the L4-5 disk space of the patient with epidural collection (blue mark), complete obliteration of the spinal canal (yellow mark), resulting from infection and inflammation of the disk space (arrowhead). There was a pseudo-fibrinous membrane over the dura and infected material in the disk space and spinal canal, all of which was removed, and the patient could walk the next day.

Correlation was sought between years of experience and other variables like the early outcome, previous surgery by the same surgeon, and complications using Pearson correlation to see if any significant association was found between any two variables. The only significant correlation was found between years of experience and previous surgery by the same surgeon, showing a weak positive correlation (r(42) = 0.321, p= 0.038). In contrast, a weak negative correlation was found between complications and early outcomes (r(42)= -0.336, p= 0.030); all correlations are outlined in Table 3. An MRI scan of a patient is shown in Figure 1, which reflects how moribund the anatomy can be, making it very difficult for an early-career neurosurgeon to intervene in such cases. 

**Table 3:** Correlations between various variables like early outcome, years of experience, previous surgery by the same surgeon, and outcome are outlined, correlation is significant at the 0.05 level (2-tailed).

		Years of experience	Early outcome	Previous surgery by the same surgeon	Complications
Years of experience	Pearson Correlation	1	0.123	0.321^*^	0.021
	Sig. (2-tailed)		0.437	0.038	0.893
Early outcome	Pearson Correlation	0.123	1	0.219	-0.336^*^
	Sig. (2-tailed)	0.437		0.163	0.030
Previous surgery same surgeon	Pearson Correlation	0.321^*^	0.219	1	-0.050
	Sig. (2-tailed)	0.038	0.163		0.753
Complications	Pearson Correlation	0.021	-0.336^*^	-0.050	1
	Sig. (2-tailed)	0.893	0.030	0.753	
	N	42	42	42	42

## Discussion

Early career surgeons should prioritize cases within their confident competence range, ensuring patient safety and building a successful track record by starting with simpler cases, allowing skill and experience growth and minimizing complications, also preventing stress overload and burnout, and fostering a sustainable work-life balance and long-term career fulfillment [8,9]. However, this is not always the case, as at times, early career neurosurgeons may come across complex procedures like RLSSs, which are more demanding and have a comparable high complication rate to those performed in fresh, unoperated spines [3,9,10]. For an early career neurosurgeon, both management of complications and patient outcome are very important, and both of these factors greatly influence his future progress and professional satisfaction, as complication management and dealing with complex cases are a matter of time and experience and are usually avoided in the early career of professional years [9].

Lumbar spine surgeries are one of the most common surgeries performed by a neurosurgeon, and among them, disk surgery has a high proportion; a majority of these surgeries are fresh surgeries, but to some extent, a neurosurgeon has to face revision spine surgeries [2,3]. The percentage of revision surgeries in our case was 10.41% (n=45), which is quite high compared to that reported in the literature and is almost as low as 3.6% by Aizawa et al. [6]. However, Cook et al. reported a high rate of about 25% amongst a population cohort of approximately 40,000 patients [10]. The majority (35/42) of revision surgeries represented only the burden of the surgeon’s operative work, and these were referred cases that did not represent the true rate of revision surgeries. The reason for this slightly high rate can be many; the hospital is the largest bedded in the province for a population of about 40 million people and is the main referral center. There was no significant difference in the mean duration of time between initial and revision surgery, whether or not the same surgeon performed it.

The most common symptoms were pain and numbness, and the most common findings on pre-operative imaging were adhesions, recurrent disk, and infection. The most commonly performed surgeries were diskectomy, decompression, and fusion, constituting 81% (n=34) of all surgeries (Table 1). This has been the fact that many patients who had an adhesion also had a small disk fragment, which was removed. Complications occurred in about 33.4% (n=14) of patients in our series, and it included dural rent in seven patients (16.7%), nerve injuries in two patients (4.8%), and Cauda equina in one patient (2.4%), and infection in four patients (9.6%). The reported rate of complications related to revision spinal surgery has been about 10-53%, including both major and minor ones, while about 21.3 % of major complications have been reported [11,12]. Incidental durotomy has been reported to be high in recurrent cases, and it ranged from 8.6% to as high as 33% in revision surgeries [10-13]. The reason behind such a high rate has been scar tissue formation and difficulty in identifying the normal structures, resulting in difficult dissection. Overt cerebrospinal fluid leak was not observed in any case, and all the rents were closed primarily with the 4/0 silk, and a drain was placed in the epidural space for 3-4 days. Infection has also been reported in four patients and was treated with antibiotics. The major complications were cauda equina and nerve injuries, which resulted in re-operation in one case and resulted in weakness in the lower limb; isolated nerve injuries were without any added neurology to the patient.

Early outcomes were excellent in 3 (7.1%), good in 26 (61.9%), and satisfactory in 10 (23.8%), one patient deteriorated after surgery, and no improvement was in two patients. Fandino et al. reported that excellent outcomes ranged from 38 to 98% depending upon the pathology, with the highest rate of a good outcome for patients with recurrent disk herniation in the adjacent level and the lowest for patients with scar tissues [11]. Similarly, Wong et al. reported a success rate of 84 % for revision spine surgeries with a minimum of two years follow-up, and bone union and mechanical instability pre-operatively were the main factors for success [13]. He also pointed out that patient selection and experience of the surgeon do matter to have a good outcome, and he attributed his high success rate to these two factors. The outcome percentage for revision lumbar surgeries is low compared to that for initial lumbar fusion surgeries, as reported by Montenegro et al. [14]. Also, the percentage of patients whose functional status declined over six months was higher in the revision group compared to the initial fusion surgery group (23% vs 12%). The factors that lead to successful revision surgery have not been uniformly agreed upon because of the many differences in patient population, operative criteria, follow-up procedures, and success criteria [15]. Fritsch et al. pointed out that the success rate significantly decreased to 20% after long-term follow-up from an initial rate of 80% in the short-term follow-up. Also, he pointed out that age and gender are not independent factors to affect the outcome [16].

The early outcome has a significantly weak negative correlation with complications, reflecting that as complications may ensue, the outcome is likely to be affected adversely; this was evident in a few cases who encountered complications, as they suffer from a neurological deficit, and though not a study variable, the cost incurred was also more than usual due to prolonged hospital stay. Cook et al. found in a large retrospective study that the overall risk of complications (dural tear, infections, pneumonia, myocardial infarction) and outcome is lower in revision spine surgeries compared to primary surgeries [10]. Apart from complications and outcomes, revision surgeries were also found to have changed unfavorable demographics, an increased proportion of co-morbidities, an increased rate of readmissions and re-operation within 30 days of surgery, and another revision within the next three months [10].

This study has many limitations, many of which are due to its retrospective nature. The patient demographics were poorly studied, including co-morbid status, obesity, hygiene status, social background, geographical locations within many miles of the primary surgeon, education status, dependency, and job status. These variables may have an impact on the follow-up visits and rate of revision surgery. 

## Conclusions

RLSSs are more complex and difficult than primary lumbar surgery due to many reasons like moribund anatomy and increased risk of complications, which may pose a difficult challenge for an early career neurosurgeon. In the early days of neurosurgical practice, such surgeries shall be dealt with extreme prudence as they may have a propensity for a low favorable outcome compared to fresh surgeries performed on the lumbar spine, due to the stated reasons, to avoid any early burnout and to have a smooth career path. 
